# Hay-Fever Caused by Vibriones

**Published:** 1870-06

**Authors:** 


### Hay-Fever Caused by Vibriones.

Helmholz says, in *Virchow's Archives*, that since 1847 he has been attacked every year, at some time between May 20th and the end of June, with a catarrh of the upper air-passages. These attacks increase rapidly in severity; violent sneezing comes on, with secretion of a thin, very irritating fluid; in a few hours there is a painful inflammation of the nose, both externally and internally; then fever, violent headache, and great prostration. This train of symptoms is sure to follow if he is exposed to the sun and heat, and is equally certain to disappear in a short time if he withdraws himself from such exposure. At the approach of cold weather these catarrhs cease. He has otherwise very little tendency to catarrhs or colds.

For five years past, at the season indicated, and only then, he has regularly succeeded in finding vibriones in his nasal secretions. They are only discernible with the immersion lens of a very good Hartmak's. The single joints, commonly isolated, are so characterized by containing four granules in a row; each two granules being more closely connected, pair-wise, and the combined length equaling 0.004 mm. The joints are also found united in rows, or in series of branches. As they are seen only in the secretion which is expelled by a violent sneeze, and not in that which trickles gradually forth, he concludes that they are probably situated in the adjoining cavities and recesses of the nose.

On reading Binz's account of the poisonous effect of quinine upon infusoria, he determined to try it in his own case. He took a saturated neutral solution of quiniæ sulph. in water=1: 740. This excites a moderate sensation of burning in the nasal mucous membrane. Lying upon his back, he dropped 4 centim. of the solution, by a pencil, into each nostril; moving his head meanwhile in all directions, to bring the fluid thoroughly into contact with the parts, until he felt it reach the œsophagus. Relief was immediate. He was able, for some hours, freely to expose himself to the heat of the sun. Three applications a day sufficed to keep him free from the catarrh, under circumstances the most unfavorable. The vibriones, also, were no longer to be found.

The experiment was made in 1867; and was repeated at the first recurrence of the attack in May, 1868, preventing the further development of the attack for that year.—*Scientific American.*

